# How Does Starch Structure Impact Amylolysis? Review of Current Strategies for Starch Digestibility Study

**DOI:** 10.3390/foods11091223

**Published:** 2022-04-24

**Authors:** Yuzi Wang, Jean-Philippe Ral, Luc Saulnier, Kamal Kansou

**Affiliations:** 1 INRAE, UR1268, Biopolymers, Interactions & Assemblies (BIA), 44316 Nantes, France; yuzi.wang@inrae.fr (Y.W.); luc.saulnier@inrae.fr (L.S.); 2 CSIRO Agriculture and Food, GPO Box 1700, Canberra, ACT 2601, Australia; jean.ral@csiro.au

**Keywords:** starch granule, in vitro digestion, experimental design, data analysis, kinetic model, starch digestion, amylase, in vitro assay

## Abstract

In vitro digestibility of starch is a common analysis in human nutrition research, and generally consists of performing the hydrolysis of starch by α-amylase in specific conditions. Similar in vitro assays are also used in other research fields, where different methods can be used. Overall, the in vitro hydrolysis of native starch is a bridge between all of these methods. In this literature review, we examine the use of amylolysis assays in recent publications investigating the complex starch structure-amylolysis relation. This review is divided in two parts: (1) a brief review of the factors influencing the hydrolysis of starch and (2) a systematic review of the experimental designs and methods used in publications for the period 2016–2020. The latter reports on starch materials, factors investigated, characterization of the starch hydrolysis kinetics and data analysis techniques. This review shows that the dominant research strategy favors the comparison between a few starch samples most frequently described through crystallinity, granule type, amylose and chain length distribution with marked characteristics. This strategy aims at circumventing the multifactorial aspect of the starch digestion mechanism by focusing on specific features. An alternative strategy relies on computational approaches such as multivariate statistical analysis and machine learning techniques to decipher the role of each factor on amylolysis. While promising to address complexity, the limited use of a computational approach can be explained by the small size of the experimental datasets in most publications. This review shows that key steps towards the production of larger datasets are already available, in particular the generalization of rapid hydrolysis assays and the development of quantification approaches for most analytical results.

## 1. Introduction

The study of starch hydrolysis by amylase, or amylolysis, in human health is central for assessing the role of starch in foods [1]. Amylolysis is also important for many industrial processes such as malting and fermentation, as well as the production of glucose, glucose syrups and bioethanol [2]. Finally, it is a fundamental natural process by which the energy stored in granular starch is delivered for the plant metabolism.

Starch comprises two polymers of glucose residues: amylose and amylopectin. Amylopectin represents by far the major fraction of native starch—75–90% of the relative dry weight of wild-type starches—while amylose is the minor fraction [3]. Both amylose and amylopectin are essentially composed of linear long chains of α-(1,4)-linked glucosyl units with α-(1,6)-branched points [4]. Amylose is essentially linear, with less than 1% α-(1,6)-branched points, while amylopectin is a highly-branched shorter α-(1,4) chain of glucosyl units with 5% α-(1,6)-branches [5]. Native starch appears in the form of granules with alternating amorphous and semi-crystalline growth rings extending from the core of the granule, which are called hilum. Starch granules exhibit a hierarchical structure in which amylose and amylopectin are arranged into a scaffolding of distinct organizational levels ranging from the molecular (~1 nm) to the granular (<100 μm) scale.

Native starch exhibits a large range of structural (e.g., macromolecular structure, relative crystallinity and crystal polymorph, granule morphology) and physicochemical (e.g., swelling, viscosity, digestibility) properties that can be used to meet various end-uses, in particular in food applications. Recent advances in crop selection and genetic approaches extend the potential properties of starch in crops even further [6]. More than ever, in vitro degradation of native starch by amylase appears as a benchmark to characterize starch from “farm to fork” before any starch-altering process (gelatinization, annealing, heat-moisture treatment, extrusion, etc.). It is especially useful for crop breeders as a marker of starch quality, which helps to orient crop selection [6]. In the human diet, starch-rich foods are digested at different rates and to different extents, which determine their nutritional value and their impacts on human health. It is generally accepted that slowly digestible starch causes a moderate postprandial glycemic response associated with beneficial effects on human health [1], while undigested starch (or resistant starch) can be regarded as a dietary or functional fiber [7] modulating the gut microbiota with beneficial health impacts. In vitro assays are commonly used to test starch digestibility, in an attempt to mimic human physiological processes. 

Hence, in vitro starch amylolysis is commonly used in different research contexts. However, whatever the research context, the in vitro assay is generally used to investigate the mechanism by which the starch structure controls its enzymatic degradation. Many publications and review papers address this topic (e.g., [8,9,10,11]), so that the effect of the observable structural features of starch on amylolysis has already been described. However, amylolysis depends on the type of enzyme used and on the interrelated structural features, including the macromolecular composition, granule morphology and non-starch components [10]. Granule size, amylose to amylopectin ratio, crystal polymorph, granular pores and channels could all affect starch degradability to different extents [12,13]. This is a multifactorial problem that involves an interdependency between the different structural levels of starch.

Different methods exist for conducting starch digestibility analysis depending on the research branch and on the pioneering works the methods are based on, such as the Englyst et al. [14] classification of starch digested fractions or the Goñi et al. [15] first-order kinetic model. This applies to the in vitro assay of amylolysis [16,17] or to the kinetic models used to describe the time-course measurements of glucose release [18]. Such a diverse offer raises questions regarding what methods are actually used and their respective benefits and shortcomings. Hence, it is necessary to report on the starch amylolysis methods (incl. data analysis) used in recent publications in a systematic five-year review, with a view to identifying suitable and consistent methods across the domains. 

The objective of this paper is to present a systematic review of recent publications investigating the relationship between amylolysis and starch structure, including starch materials, factors investigated, in vitro assay techniques and data analysis techniques. The review provides an overview of dominant research strategies, bottlenecks and emerging strategies in view to outline promising methods for a benchmark starch digestibility analysis. This review paper has two main sections; (i) the first section presents an overview of the intrinsic starch structural features and the experimental conditions that influence the native starch amylolysis results, and (ii) the second section presents a systematic review of the publications on the topic from 2016 to 2020. 

## 2. Factors Influencing Starch Hydrolysis

### 2.1. Starch Structure—Amylolysis

The processing of native starch generally affects its susceptibility to enzymatic degradation significantly, to the point that it can get more important than the native structure; here we used “starch” to refer to native starch unless specified otherwise. 

The starch enzymatic hydrolysis is a heterogeneous reaction as the catalytic act takes place at the interface between the solid phase (starch) and the liquid phase that conveys the enzyme. It involves different steps, including the initial diffusion of enzyme through the different levels of starch organisation followed by the adsorption on the surface (formation of enzyme-starch complex) and finally the hydrolytic [19]. Different types of enzymes such as glucosidase, isoamylase, glycosyl-transferase, β-amylase and α-amylase can catalyze the chemical degradation of starch. Alpha-amylase, the most common amylolytic enzyme, catalyzes the hydrolysis of α-(1,4) glycosidic bonds in starch polymers in an endo-acting fashion [20]. Alpha-amylases (E.C.3.2.1.1) isolated from pancreas or salivary glands are widely used in starch digestion research studies, often in combination with fungal amyloglucosidase [11] to convert the amylolysis products into glucose. Various methods have been proposed to monitor and to interpret the enzymatic digestion of starch. For a long time, Englyst’s method [14] has been adopted for assessing “in vitro” starch digestion. This method distinguishes three starch fractions—RDS (Rapid Digested Starch), SDS (Slow Digested Starch) and RS (Resistant Starch)—based on the amount digested within, respectively, 20 min, 120 min and remaining after 120 min. Besides this, fitting the time-course measurements of the starch enzymatic degradation with a kinetics model is another method that has become quite common, which presents clear interest in term of data analysis [18]. 

The factors determining the enzymatic susceptibility of native or processed starch (e.g., cooked) have been investigated extensively. While starch is generally processed in human food, native starch still raises much scientific interest as a natural substrate for most amylolytic enzymes. In addition, the combination of multiple processed methods with water, high temperature, milling, etc., associated with the starch structural parameters generate a very complex analysis with endless sources of variability [21].

Native starch is present in the form of granules made of concentric alternating amorphous and semi-crystalline growth rings of carbohydrate polymers. While, this general organization is common across the plant kingdom, the overall shape size and the polymers’ organizations vary greatly with the botanical origin [22]. 

The starch granule can be described through four organizational levels: (i) the chemical structure of starch polysaccharide constituents (~1 nm), (ii) the crystalline and lamellar structure (~9 nm), (iii) the growth rings (120~500 nm) and (iv) the granular level (1~100 μm). The relations between starch structural features and the enzymatic hydrolysis are briefly described in this section, starting from the granular level to the chemical structure of the polymers. The aim was to provide a broad overview; the additional and thorough information can be found in several dedicated review papers [8,9,10].

#### 2.1.1. Granule Morphology 

The starch granule characteristics account for a significant part of the amylolysis susceptibility [5,23,24]. Starch granule diameter commonly ranges from 1 μm to 100 μm [23], while some could even be less than 1 μm across the plant kingdom [25]. The granule size distribution can be either unimodal, with a single predominant granule type, for example in maize, potato and pea, or bimodal, with two granule types or more, for example in barley, wheat and oat [5]. The starch granule average diameter is a few thousand times larger than an α-amylase with a 4 nm hydrodynamic radius and offers many surface binding sites to the α-amylase [26]. For many starches, however, the granule size is negatively related to the amylolysis susceptibility, indicating a limiting surface adsorption (or binding) for the amylase [13,24]. There are many exceptions to this relation, and in general the granule size alone is not sufficient to account for the differences of susceptibility to amylolysis across the botanic sources [24,26,27,28]. Thus, the granule surface characteristics, such as smoothness or the ratio of ordered-disordered glucan chains, also impact the adsorption of amylase, especially in the early stages of hydrolysis [29,30,31]. 

Many cereal starches granules, such as maize and rice starch, exhibit surface pores of 0.1–0.3 μm in diameter and radially oriented interior channels of 0.07–0.1 µm in diameter, which extend the surface available for the adsorption of enzyme as these pores are believed to be initial openings and entry points for the enzymes [32,33]. Hence, starch granules with many pores, channels and cavities present a high internal surface that favors the binding of enzymes, thus reducing greatly the impact of the granule size [13]. 

The presence of pores and channels results in an ‘endo-corrosion’ (from inside out) digestion pattern, where α-amylase creates and enlarges pits from the core to the surface [16,33]. Granules without pores, as for potato or amylomaize starch, are digested from the surface by ‘exo-corrosion’ (from outside in) and at a much lower rate than by endo-corrosion. While the granule morphology controls the binding areas access, the local conformation and glucan chains packing inside the granule is assumed to determine the effectiveness of the α-amylase hydrolytic action [11].

#### 2.1.2. Starch Polymers Arrangements Inside the Granule 

Native starch granules are layered structures made of crystalline regions with orderly packing of amylopectin chains double-helices and amorphous regions made of amylose and amylopectin without detectable molecular ordering [11]. An x-ray diffraction method is used to identify the type of crystalline structure and, quite often, to quantify the crystalline/amorphous structure ratio in the starch sample. Crystalline regions are generally considered less digestible than amorphous layers. However, some contradictory evidence supports equivalent digestion rates for the semi-crystalline and the amorphous growth rings [16,34,35]. Zhang et al. [16] described a side-by-side digestion mechanism for endo-corroded granules, suggesting that the local molecular density of the amorphous rings could be as rate-limiting as the semi-crystalline layers.

There are two basic polymorphic types of crystalline structures in native starch, the A-type encountered mainly in cereal starches and the B-type found in potato or high amylose maize variants. A-type crystallites are made of double-helices of amylopectin chains ordered in a monoclinic unit cell, while B-type crystallites are made of double-helices ordered in a hexagonal unit [5]. An additional C-type crystal that combines A-type and B-type can be found in the starch from many legumes. Many studies have reported the higher degradability of A-type starches compared to B-type starches even across botanical sources [28,36,37,38]. B-type crystals are more prone to hydration than A-type, which would favour a higher hydrolysis rate. Indeed, while A-type starch crystal contains eight water molecules in each tightly packed helix, the B-type crystallites have 36 water molecules in each unit [5]. Thus, A-type crystallinity seems to affect more specifically the hydrolysis kinetic in the early stage, while B-type crystallinity is often remarkably related to the final extent of the hydrolysis [36,37,39].

In native starch, the distribution of amylopectin chains, represented by unit Chain Length Distribution (CLD) or the short:long amylopectin side-chains ratio, is generally well correlated with the starch hydrolysis [28,40,41]. The CLD is often determined by chromatographic separation (fluorescence-assisted capillary electrophoresis after enzymatic debranching by isoamylase), and the chains are generally classified as short chains below a degree of polymerization of 36 glucose residues and as long chains beyond that [22]. The CLD is often correlated with other structural functional factors of starch. In particular, the CLD profile is characteristic of the botanical origin, as A-type crystallites are made of shorter chains than B-type crystallites [34,41]. The latter correlation is believed to be key for explaining the higher susceptibility of A-type over B-Type crystals to amylolysis. 

The ratio amylose/amylopectin is usually negatively correlated with the susceptibility to amylolysis, which is counter-intuitive as the α-1,6 linkages of the amylopectin should hamper the binding of α-amylase [42]. Lopez-Rubio, Flanagan, Gilbert and Gidley [43] showed that amylose chains can rearrange into enzyme-resistant structures of high crystallinity during amylolysis, and it is suggested that amylose contributes to the increased resistance of high-amylose maize starch in the granule structure, making it less prone to the α-amylase action [8]. The role of amylose in amylolysis remains nonetheless unclear in the literature, as an extreme value for amylose content is often correlated with other distinctive features such as B-type crystallinity, variation in amylopectin CLD and lack of pores at the granule surface described in high-amylose maize starch [28,37,39,43].

#### 2.1.3. Starch Endogenous Proteins and Lipids 

Besides amylose and amylopectin, purified starch contains additional minor components, mainly proteins and lipids associated with the starch granule located on the surface or imbedded within the native starch granules [44,45,46,47]. Baldwin [48] reports the presence of ~0.25% protein and up to 1.0% lipid for typical washed cereal starch and 0.05% protein and 0.05–0.1% lipid for root or tuber starch. Participating in the surface structure, lipids and proteins can affect the enzyme susceptibility as well as other functionalities of starch despite their relatively minor proportions [49]. Hence, surface proteins and lipids could reduce the enzyme binding by blocking the adsorption sites as the defatting and the removal of granule-associated proteins enhances digestibility [50,51]. Surface lipid and proteins that can accumulate randomly at the surface of the granule depending on the varieties can also represent an additional layer of resistance for enzymes to reach [52]. 

Starch lipids present a high diversity. They can be detected as Free Fatty acid (FFA), or lipids can form amylose-lipid complexes in native starch amorphous or crystalline arrangements, i.e., V-type crystallinity [53]. An amylose-lipid complex may limit the enzyme accessibility to the substrate by restricting the starch swelling power, which, in turn, would limit the water uptake and the amylose leaching out [54,55].

### 2.2. Amylolysis Conditions 

While starch structure and composition are universally recognized for influencing amylolysis, the actual conditions defining the reactions can have a significant additional impact on the hydrolysis results. 

**Substrate concentration**. According to Tawil et al. [35], both the reaction rate and the final hydrolysis extent are significantly reduced at high starch concentrations (from 50 mg/mL dry basis and onwards for maize starch). The negative impact of high starch concentrations on amylolysis are likely due to several factors, including restricted enzyme diffusion and consequently increased auto-formation of enzyme-resistant structure during hydrolysis, e.g., B-type or V-type crystallites [35,43]. 

**Source and concentration of enzyme.** Enzyme type, biological origin and concentration influence the kinetic and hydrolysis final extent as well as the degradation products. The α-amylases particularly have diverse modes of actions and product specificities depending on their origin [42,56]. For example, Porcine Pancreatic α-amylase (PPA) produces primarily malto-oligosaccharides—maltose, maltotriose and maltohexaose—[56] while *Bacillus licheniformis* α-amylase, extensively used in industrial processes, produces mainly maltopentose [57]. The difference between α-amylase modi operandi could be explained by variations in the substrate binding or catalytic sites, as well as in the thermolability or purification process [58]. For in vitro starch digestion assays, α-amylase is often combined with Amyloglucosidase (AMG) (or glucoamylase) [59] as a substitute for the exo-acting α-glucosidase involved in mammalian digestion. AMG’s intended role is mainly to hydrolyze the soluble oligomers produced by the α-amylase action to release glucose or maltose units. These two types of enzymes can be combined and used in a one-stage assay, or consecutively, in a two-stage assay. When used in combination, they display synergistic activity on native starch as α-amylase hydrolyses the large molecules, thus providing substances to AMG, and AMG enhances the α-amylase action by hydrolyzing inhibitory oligosaccharides into glucose and by detangling double helical structures [59]. 

**Other experimental conditions**. As for any enzymatic reaction, the amylolysis must be conducted within a range of incubation temperatures and pHs that would ensure optimal enzymatic activity. The incubation temperature and duration need to accommodate with the nature of the substrate, as modification starch granule structure, especially starch annealing and starch gelatinization, may occur in the conditions of a hydrolysis assay [60]. Other oversighted factors such as the stirring conditions (speed and probably the stirring mode) could also be considered as parameters affecting the hydrolysis rate. Indeed, Roldan-Cruz, Garcia-Hernandez, Alvarez-Ramirez and Vernon-Carter [61] showed that the activity of PPA is enhanced at a low stirring speed (0–250 rpm) but decreases significantly when the stirring speed is elevated (250–1500 rpm). They suggested that intense stirring can alter the enzyme structure or could impair the enzyme-substrate binding.

### 2.3. Current Limitations and Challenges

Observational studies of starch degradation during hydrolysis have greatly contributed to our current understanding of the mechanisms. The development of new analytical techniques allows scientists to monitor continuously the dynamic changes of the starch granule during the course of amylolysis [44,62]. This research relies mainly on the deployed analytical techniques and involves generally only a few starches with very distinct features, e.g., high-amylose starch vs no-amylose starch. While crucial for describing the overall processes, focusing on extreme structural features has some limitations. Using a strong dominant mutation that severely impacts starch structure can overlook the range of fine structural alterations seen in more conventional starches, thus hindering our ability to understand the role of each feature both individually or in combination. Amylose content is an example of strong drive, as the zero-amylose mutation does not only suppress 25–30% of the total starch composition (by removing amylose) but also dramatically impacts the amylopectin chain length structure, crystallinity, overall granule structure and granule size distribution, as well as the endogenous lipid and protein contents. That “tree that hides the forest” is a perfect example on how a unique mutation would drive most of the changes observed and therefore would provide only limited information on the mechanistic underlying amylolysis of conventional starches. Most of the conventional starches displayed only a slight variation in chain lengths, lipid content or slightly smaller granule size due to small genetic iterations or environmental changes. 

Deciphering the impact of each and every factor affecting the amylolysis profile is critical but challenging when those factors synergistically interact. Potato starch, a tuber starch, has rather long amylopectin side-chains, B-type crystallinity and large-size granules without visible surface pores while rice starch, a cereal starch, presents an almost opposite profile. All the potato starch features can account for the low susceptibility to amylolysis (not mentioning phosphate groups bound to amylopectin) and, vice-versa, for the high susceptibility of rice starch. 

Hence, determining to which extent each of these factors individually or synergistically accounts for the overall susceptibility to the enzymatic degradation is challenging. Besides this, factors measured in the context of a research study captures only a fraction of the real starch structure. A significant fraction remains unknown (Kansou et al., 2015b). Hence, the starch structure-amylolysis conundrum is a multi-dimensional problem affected by uncertainty due to unknown parameters. 

Purely observational investigations have limitations in addressing this type of problem, and other research orientations are proposed, such as the identification of dominant rate-determining factors of the amylolysis [10,11,63]. 

The following section reports on the experimental designs and analysis employed by recent studies to address the starch structure-amylolysis conundrum. 

## 3. Systematic Review of Papers from 2016–2020

### 3.1. Scope and Search Protocol

**Figure 1:**
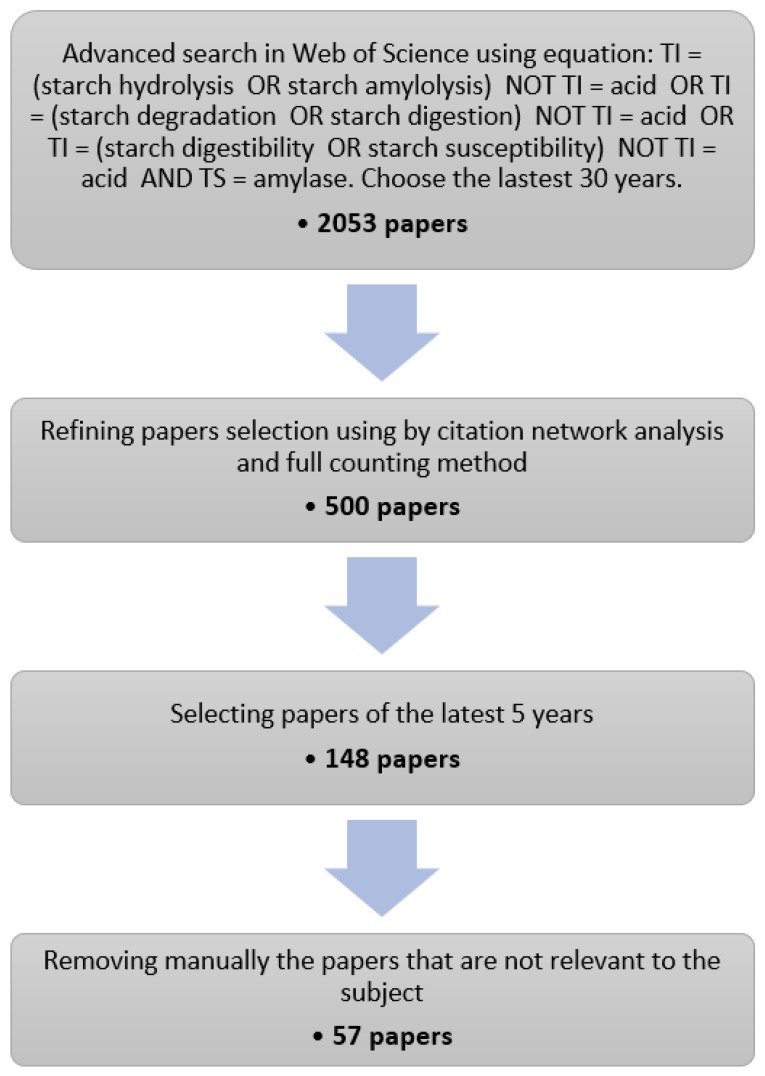
Flow chart for selecting relevant papers. TI and TS stand respectively for title and topic. While we have generally respected the PRISMA guidelines, several items related to data-extraction, meta-analysis and to the involvement of potentially many reviewers are not applicable for this work.

Starch enzymatic degradation has been investigated for decades with continuing interest since 2016. Using a literature research engine (Web of Science, WoS) and the procedure described in Figure 1, 57 articles published between 2016 and 2020 on this topic have been selected. The results of the selection procedure as well as the information extracted for each article are supplied as Supplementary Materials. 

**Figure A1:**
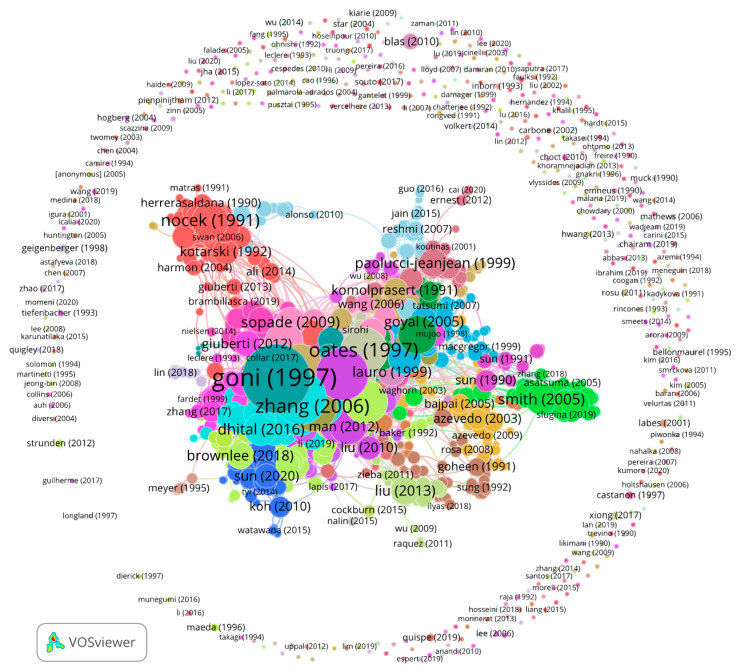
Citation network visualization of 2053 articles obtained from the WoS with 6178 links. The ring around the network core represents 509 articles with no links to the other articles. The article with the highest number of links (161) is Goñi et al., (1997), labelled goni (1997). Size of node reflects the number of links.

**Figure A2:**
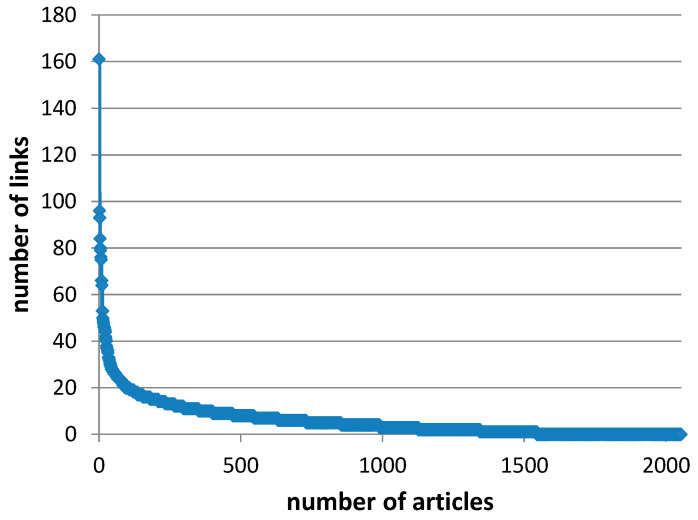
Number of links, i.e., inter-citations, per article for the 2053 articles obtained from the WoS.

**Figure A3:**
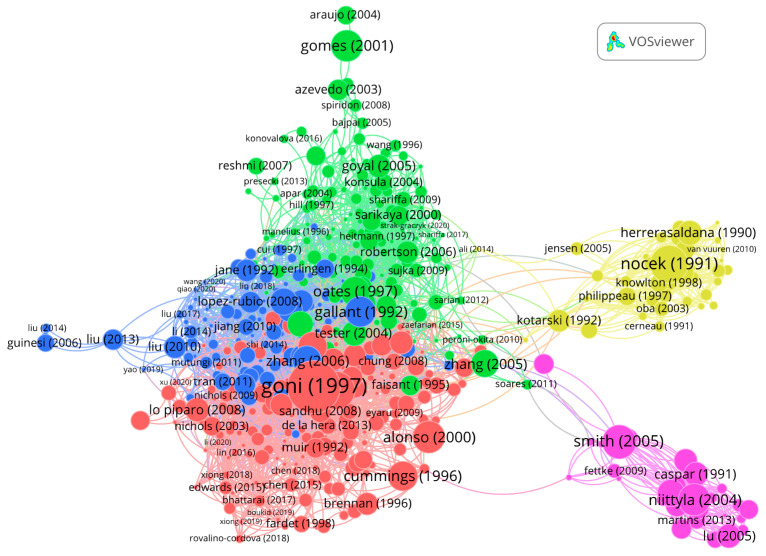
Citation network visualization of the 500 articles of the panel with 3195 links and 5 clusters. The article with the highest number of links (96) is Goñi et al., (1997). Size of nodes reflects the number of citations.

In the first stage of the selection procedure, a list of 2053 journal articles covering the last 30 years was obtained from the WoS using a general research equation about starch hydrolysis by α-amylase, explicitly excluding acid hydrolysis (Figure 1). Stage 2 aimed at narrowing down this list to articles that were part of the major citation network on the topic. To this end, we performed a citation network analysis using a dedicated tool (VOSviewer, [64]). Citation networks are made of nodes, which represent articles, edges or links, which represent inter-citations between the articles. From this information, VOSviewer computes a distance-based map where the distance between two articles reflects their similarity, i.e., the citations they have in common. From the list of 2053 articles, we produced a complete citation map of 6178 links and a distribution of the number of inter-citations per article (Figure A1 and Figure A2). The distribution follows a steep decreasing curve, and 509 articles have no links with the others, it is also visible that the network is a “dense” core group of articles. Given the distribution, we estimated that a set of 500 articles was large enough to capture a consistent group of interlinked articles on the topic, while having a fair representation of the ongoing research. Using VOSviewer again, we selected a set of 500 articles with the highest similarity level, starting from Goñi et al. [15], which is the article with the highest number of links as well as a reference on the topic. The resulting citation map is provided in Figure A3. It has 3195 links and only two articles without links, which indicates that we succeeded in sampling the “densest” part of the network. In the final stages, we retained the publications of the last five years and manually removed the articles not in the scope. 

The information about the 57 articles of the panel used for the following analysis is supplied as an excel table; in the Supplementary Materials, each article is assigned an id with the name of the first author, the year of publication and, when needed, a letter (‘a’, ‘b’…) to distinguish between articles with same name that were published in the same year.

Table 1 reports the topics addressed by the panel. The large majority (70%) focused on the factors and mechanisms influencing starch amylolysis. The remaining papers described the effect of modification treatments on starch structure and amylolysis to meet various end-product performances. The modifications included an amylolysis optimization process involving novel degrading enzymes as well as comparing and developing different kinetic models to improve amylolysis analysis.

### 3.2. Type and Size of Samples 

Cereal starch from maize, rice and wheat, together with potato starch, were extensively studied in our publication panel (Table 2), most likely as ingredients available worldwide. Maize starch was the most frequently investigated among crops (35% of the papers); it includes the starch from well-known commercial lines of maize mutants, e.g., *wx* (waxy), *ae* (amylose-extender) and *su2* (sugary), with various granular and macromolecular structures [65]. Other starch types, including cassava sweet potato, bean and pea starch, complete the publication list. 

Table 2 also provides the number of papers that compare wild-type, mutant, transgenic lines and single-segment substitution lines starch, all with a similar genetic background. This type of experiments is reported in 14% of the panel and involves mainly rice (Table 2). Surprisingly while maize mutant starch is present in many publications, the panel does not include specific works on maize genetic variants. This suggests that this crop has been already well covered in past publications. Starch obtained from genetic variants presents structural alterations caused by either deletion of or modifications of the expression (silencing, downregulation, upregulation) in the enzymes of the starch biosynthesis pathway. Those enzymes are generally acting on the elongation, the branching or the debranching of the starch polymers [66]. Genetic variants introduce targeted starch structural modifications and often generate near isogenic control lines, allowing comparative studies focusing primarily on the altered features [37,63]. 

**Figure 2:**
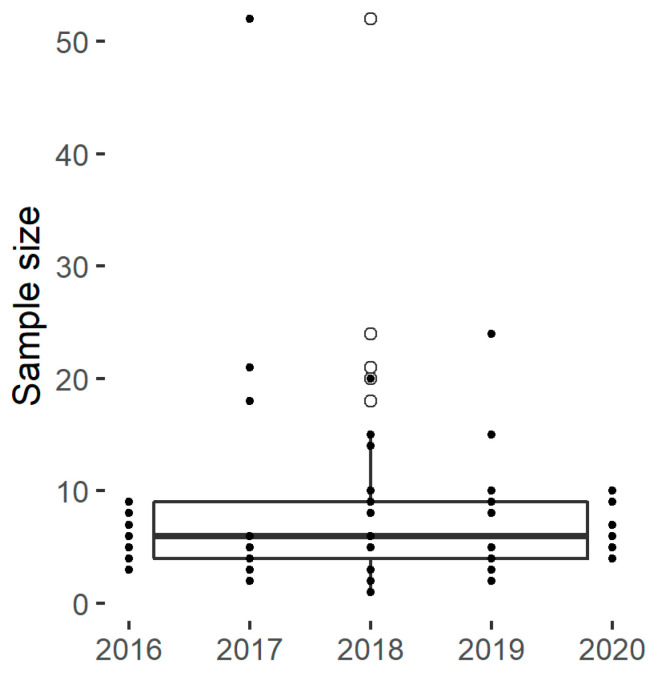
The sample size for selected papers.

The experimental designs found within the panel include on average eight distinct samples (Figure 2), which allows the investigation of a limited number of factors. In practice, the number of samples is limited by the availability of starch materials with desirable characteristics and by the experimental resources required to characterize the samples or to run the hydrolysis assays. Hence, many publications included a selective number of diverse samples with marked characteristics and/or innovative structural or functional characterization techniques. An alternative approach was to analyse a higher number of samples using established analytical techniques and hydrolysis assays, as for Martens et al. [28,67]. These authors reported the results of a retrospective power analysis to confirm that the number of samples were sufficiently large to reveal the discrepancies between the degradation kinetics.

### 3.3. Characterisation of Starch Structure

**Figure 3:**
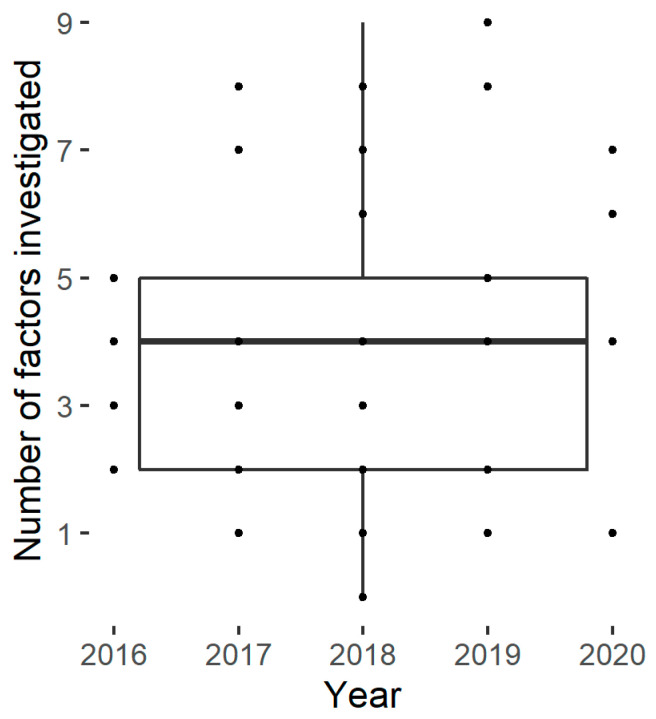
Number of factors studied by the selected papers.

The number of studied factors described in the publication panel varies from one to nine, with an average of four (Figure 3). No trend could be identified in the number of factors investigated over the last five years. 

The most frequent factors describe distinct organizational levels of native starch (from granular to molecular level) as well as few functional properties (Table 3). At the molecular level, amylopectin chain length distribution and amylose content were both measured in 32% of the panel. However, as mentioned in Section 2.1, the impact of amylose content on amylolysis is still unclear, and a few works of the panel observed an impact, e.g., Kuang, Xu, Wang, Zhou & Liu [68] and Lin et al. [12], while others did not, e.g., Martens et al. [28]. The amylose content is commonly estimated with the iodine binding colorimetric method [69], even though this method can overestimate the amount of amylose due to the formation of amylopectin-iodine complexes [70]. Therefore, alternatives, e.g., concanavalin A precipitation and the size-exclusion chromatography weight distributions, are increasingly applied for a more accurate estimation [12,28,71,72,73]. Investigations of minor non-starch components are reported in 17% of the panel. This concerns especially the proteins either via the protein content estimation using nitrogen estimation by the Kjeldahl method, the Dumas method, a nitrogen analyzer [71,74,75] or by comparing the hydrolysis results before and after protein removal (e.g., [51]). At the crystalline and lamellar levels, the crystallinity (type and quantification) measured by X-ray diffraction appears to be the most studied feature (53% of the panel), certainly due to the fact that the starch crystallinity is often effectively correlated to physical, mechanical and technological properties and of course to amylolysis [43]. The lamellar structure, described through lamellar ordering and thickness using small-angle X-ray scattering, is also reported in 23% of the panel. At the granular level, 37% of the panel includes a granule morphology analysis by Scanning Electron Microscopy (SEM). SEM is used to determine the size and shape of the granules, as well as their surface properties, number of pores, size and dispersity and degradation signs [28,76,77]. The granule size distribution, usually determined by Laser Diffraction Sizing (LDS), is present in only 19% of the panel. 

The functional properties reported in the panel publications are mainly related to starch gelatinization (28%) measured by Differential Scanning Calorimetry (DSC). DSC reflects on the swelling, the leaching of soluble polysaccharides, the disruption of the molecular order and the melting of crystallites [78], thus providing a compelling understanding of starch gelatinization properties. In comparison, results from the Rapid Visco Analyzer (RVA) (pasting properties in Table 3) are much less common (<9%), possibly because RVA requires a significant amount of biological material for a single analysis and it has a rather low throughput. 

Each of the uncommon factors (“others” in the Table 3) had been measured in less than five studies in the panel. They can describe the starch intrinsic structure, e.g., molecular weight distribution and endogenous lipid content, or extrinsic factors, e.g., enzyme and substrate concentrations, temperature, incubation time, plant genetics and environment. 

### 3.4. Characterisation of Hydrolysis Kinetics

In most publications, the extent of starch enzymatic hydrolysis is determined by an enzymatic assay quantifying the glucose release. The most frequent method, with 51% of the panel, is the glucose oxidase and peroxidase (GOPOD) assay [79] (Table 4). It has high specificity but lacks relative stability over time and has a relatively high cost per sample [80]. The measurement of reducing ends by dinitrosalicylic acid (DNS) [81] and by 4-hydroxybenzoic acid hydrazide (PAHBAH) colorimetric methods are alternative options to glucose assays that are simple to apply with relatively low cost. This family of dosage techniques is in general much more rapid and easier to handle than the dosage of total solubilized sugars by the orcinol–H_2_SO_4_ method used in older works (e.g., in [37]). Nevertheless, with equimolar amounts of maltodextrins, the response of the reducing sugar by the DNS method increased with the increasing number of glucose units [82], which leads to an overestimation of the hydrolysis extent. 

**Figure 4:**
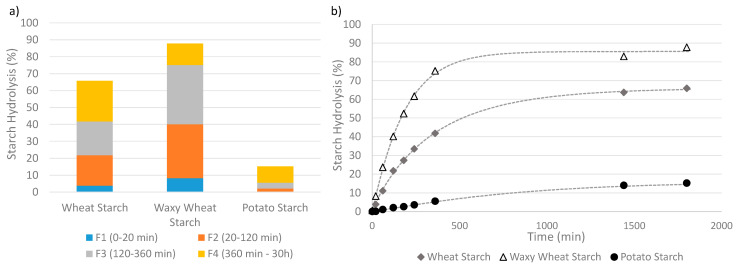
Two ways of analyzing degradability, applied to wheat starch, waxy (no-amylose) wheat starch, potato starch. (**a**) Identification of hydrolysable fractions of growing susceptibility to hydrolysis. The non-hydrolysable or Resistant fraction is the difference between the total amount of starch and the sum of all the digested fractions. Results are only for illustration as the data was not obtained from a standard in vitro digestion procedure. (**b**) Time-course measurements of the hydrolysis kinetics described by a fitted Weibull function.

For 91% of the panel, an enzymatic assay is used to obtain kinetic data of the starch degradation by an α-amylase (30%), sometimes in association with an amyloglucosidase (61%). Several methods were used to interpret the kinetic data. Twelve percent of the panel used a starch classification method, more precisely derived from Englyst’s method, to identify starch fractions with decreasing digestibility based on their digestion time [14]. Figure 4a presents an example of the classification of digested starch from three distinct sources into different fractions of increasing digestibility level. This approach basically requires one measurement of the hydrolysis extent per fraction. Identification of Resistant Starch (RS) content via the AACC (American Association of Cereal Chemists)-approved Method, which separates the RS from the non-resistant starch and then hydrolyzes this fraction into glucose using AMG, was used by Uriarte-Aceves et al. [71], whereas the quantification via the AOAC (Association of Official Agricultural Chemists) Method [83] was not reported in the panel. Eighty-eight percent of the panel described the starch degradation over time using a kinetic model fitted to the data, which appears to be a common practice to quantitatively analyze the kinetics parameters [18]. A simple kinetic model can describe not only the degradability level at different times but also the shape of the curve describing the hydrolysis time course and the reaction rate coefficient. Figure 4b presents examples of time-course measurements of the hydrolysis extents fitted with a modified first-order kinetic model, i.e., the Weibull model, Equation (6).

Seventy percent of the panel applied the empirical first-order kinetics (Table 5) using Equation (1) proposed by Goñi, Garcia-Alonso & Saura-Calixto [15]:

(1) $$C=C_{\infty }\left(1-e^{-kt}\right)$$

where C is the product concentration at time ***t***, $C_{\infty }$ is the production concentration at the end of the hydrolysis and ***k*** is the reaction rate constant. Although the first-order kinetics describes well the hydrolysis profile, it needs an accurate estimation of $C_{\infty }$, which can be difficult to achieve even with a prolonged hydrolysis assay [84]. The application of the log of slope (LOS) plot via equation:

(2) $$\ln\left(\frac{dC}{dt}\right)=\ln\left(C_{\infty }k\right)-kt$$

by Butterworth et al. [84] allows the calculation of $C_{\infty }$ from the intercept on the y axis that could avoid the aforementioned issue. Forty-six percent of the panel applied the LOS plot for obtaining a more reliable estimation of $C_{\infty }$ and ***k***. 

Kinetics data do not always follow a first-order kinetics, which led to adaptations of Equations (1) and (2). Two main adaptations of the first-order kinetics model have been proposed to obtain a better fitting of kinetic data, a sequential model that describes a fraction of the starch digested before others and a parallel model that describes two or more fractions of starch digested simultaneously at different rates [85]. Edwards, Maillot, Parker and Warren [86] applied a sequential first-order kinetic model (Equation (3)), whereas Li et al. [85] and Bello-perez, Agama-Acevedo, Garcia-Valle and Alvarez-Ramirez [87] applied variants of parallel first-order kinetic models, respectively Equations (4) and (5):

(3) $$C_{t}=\left\{\begin{array}{c} C_{1\infty }\left(1-e^{-k_{1}t}\right),\;\;\;\;\;\;\;\;\;\;\;\;\;\;\;\;\;\;\;\;\;\;\;\;\;\;if\;t\leq t_{int}\\ C_{int}+C_{2\infty }\left(1-e^{-k_{2}\left(t-t_{int}\right)}\right),\;if\;t\geq t_{int} \end{array}\right.$$

(4) $$C_{t}=C_{0}+C_{1}\left(1-e^{-k_{1}t}\right)+C_{2}\left(1-e^{-k_{2}t}\right)$$

(5) $$Y_{t}=Y_{RS}+Y_{RDS}e^{-k_{RDS}t}+Y_{SDS}e^{-k_{SDS}t}$$

where $t_{int}$ is the time of intersection of two consecutive reactions; $C_{int}$ is the concentration of product at $t_{int}$; $k_{1}$ and $k_{2}$ are the corresponding rate constants for each type of starch; $C_{0}$ is the starting concentration of digestible starch; $C_{1}\;$ and $C_{2}$ are concentrations of two digestible starch types with distinct susceptibilities to amylolysis. 

*Y_**t**_* is the normalized starch concentration and $Y_{RS}$, $Y_{RDS}$ and $Y_{SDS}$ are the concentrations of *RS*, *RDS* and *SDS*; $k_{RDS}$ and $k_{SDS}$ are the rate constants of *RDS* and *SDS*. Compared to the sequential model, the parallel model avoids breakpoints at the times of intersection between two hydrolysis phases [85].

A Weibull model proposed by Kansou, Buléon, Gérard & Rolland-Sabaté [88] is another variant of the first-order kinetics model (Equation (6)). In the panel, it has been applied by Olawoye & Gbadamosi [89] to obtain an accurate fitting of biphasic hydrolyses. In this model the reaction rate-related parameter ***k*** is affected by a parameter *h* that reflects the time-dependency of the reaction rate coefficient:

(6) $$C=C_{\infty }\left(1-e^{-kt^{1-h}}\right)$$

### 3.5. Statistical Analysis of Starch Structure-Amylolysis Relationships

The majority of the panel publications employed standard statistical analysis methods (Table 6). Up to 72% present the results generated using ANOVA, often followed by the result of a test distinguishing the different groups, e.g., the Tukey test, 19% computed linear correlations (Pearson) or the most robust rank correlation (Spearman) that assesses monotonic relations between two variables. Multivariate statistical analyses are reported in only 7% of the panel (Table 6). Principal component analysis (PCA) is the most common multivariate analysis, it is used to identify the multilinear relations between several factors via a new set of orthogonal variables, so-called principal components. PCA allows the relation of combinations of interacting factors such as granule diameter, pores, crystalline type and side-chain length of amylopectin with kinetic parameters of the amylolysis (e.g., [28,39]).

The conditions of application of the usual linear models, including the linear regression models or the ANOVA, are often problematic in the context of starch structure-degradation studies because of the collinearity of the data [39]. Despite this limitation, the relatively small size of the datasets envisioned in Section 3.2 and Section 3.3 hinders the application of multifactorial analysis or clustering techniques alike.

## 4. Conclusions and Perspective

Despite many publications, the study of “structure-degradability” relationships remains challenging, as the starch hierarchical structure is complex and multidimensional and our understanding is incomplete. The interdependencies between the structural features of starch hinder the identification of their respective contribution to the enzymatic degradation. How is this challenge currently addressed by the scientific research? 

To answer this question, our systematic review of a panel of 57 recent publications on this topic outlines the following aspects of the experimental research:

- Predominant sources of starch are a few major starch-rich crops of the human diet: cereals (rice, maize, wheat) and tuber (potato). Focusing on selected crops limits the splitting of the research effort and favors the understanding by accumulating knowledge.
- A typical starch-degradability study investigates the digestibility of eight different samples via four structural/functional factors characterizing the starch. The most frequent factors in the panel involve the crystallinity, the granule morphology via SEM, the amylose content, the chain length distribution and the gelatinization properties. The selected starch materials present marked distinctive characteristics, such as high amylose vs. wild-type vs. no-amylose starches, the presence or absence of granule pores and A-type vs. B-type crystalline polymorph starches. On the one hand, this generates contrasted digestibility profiles, while on the other hand, this tends to overlook the role of the accompanying structural alterations, which are possibly critical to discriminate amongst conventional starches.
- The size of the average experimental dataset (i.e., starch materials × measured factors) might be insufficient to exploit recent data analysis techniques (e.g., machine learning techniques). This is a limitation to addressing complexity, as computational approaches are necessary to address multifactorial systems.
- Quantitative approaches exist for analyzing most structural factors of starch. Even though quantification can be indirect and uncertain—the determination of crystalline proportion from X-ray diffractometers, the quantification of granule characteristics from SEM and the continuing development of quantitative approaches is an important lever to generalize the use of computational techniques.
- Several assays and methods can be employed for the measurement and the analysis of starch hydrolysis, in relation to the research topics. While diversity in this matter is necessary—studying starch digestibility in human diet is different from investigating the action of a specific amylase—using the same method for a given topic can favour the comparison of inter-publications. This review presents the most frequent assays and methods used in recent works; this can guide the selection of methods in future studies.

Overall, this review shows that the dominant strategy for conducting starch digestibility analysis is to circumvent the complexity of the underlying mechanism by careful selection of contrasted substrates. The most promising works in that regard use mutants and transgenic lines from a generic line to obtain starch samples displaying variations for specific targeted parameters, thus limiting the variability for unknown parameters. A second strategy that is in the minority so far addresses the interrelated factors issue using multivariate analysis techniques. Both approaches have drawbacks: the former requires more efforts from the plant genetic domain to produce starch with interesting characteristics and depends on a forward genetic approach, while the latter demands larger datasets in terms of number of samples and number of measured factors. 

The second strategy appears closer at hand for the research community, as the average size of the datasets will naturally increase with the development of high-throughput techniques and the growing quantification capability of the analytical results. This review has shown that key steps towards scaling up experimental designs are already available, in particular the generalization of rapid hydrolysis assays with the dosage of reducing sugars and the application of kinetic models to interpret the data. These combined would provide a reliable quantification and analysis of the amylolysis profile. A remaining bottleneck is certainly the development of high-throughput relevant analytical techniques to describe the starch properties. 

## Figures and Tables

**Table 1:** Selected articles from 2016 to 2020 sorted by topic.

Topics	Nb. of Publications	Id of the Publications *
Influencing factors and mechanisms of starch amylolysis	40	huang2016, lin2016a, lin2018, martens2018, nhan2017, li2020a, qiao2019a, qiao2019b, qiao2020, li2018, xu2017, chen2016b, edwards2018, guo2018b, kuang2016, teng2016, yao2019, uriarte-aceves2018, teng2019, liu2019, lan2016b, li2020c, villas-boas2019, guo2017a, guo2018a, yu2018b, chen2016a, guo2016, qiao2017, guo2017b, li2020d, hu2018, ma2020a, ma2020b, yu2018a, takagi2018, vernon-carter2019, liu2018, martens2019, hargono2018
Effect of modification treatment on starch structure and amylolysis	11	shariffa2017, akanbi2019, anderson2016, qiao2016, kim2017, wang2017a, wang2019, benavent-gil2017, shah2018, shi2018, yang2019
Optimization of starch amylolysis	3	slavic2016, das2019, peng2018b
Analysis of starch amylolysis	3	bello-perez2018, li2019a, olawoye2020

* Supplementary Materials table provides information for the publication with the corresponding id.

**Table 2:** Type of sample used in the panel of papers.

Starch Botanical Origin	Count	
	Total	Incl. Genetic Variants Analysis
maize	20	0
rice	14	5
potato	12	0
wheat	8	2
bean	5	0
cassava	5	0
pea	4	1
sweet potato	4	0
lotus	4	0
others	29	0

**Table 3:** Factors investigated by the selected papers.

Structure Level	Investigated Factors	Count
Molecular level	chain length distribution	18
	amylose content	18
	protein content	8
	molecular size distribution	7
	molecular order	7
Crystalline and lamellar level	crystallinity	30
	lamellar structure	13
Granular level	granule morphology	21
	granule size distribution	11
Functional properties	gelatinization properties (DSC)	16
	pasting properties (RVA)	5
Other	others	38

**Table 4:** Sugar measurements for selected papers.

Sugar Measurement	Count
GOPOD	29
DNS	9
PAHBAH	8
Anthrone-H_2_SO_4_	5
others	8

**Table 5:** Kinetic models and the corresponding kinetic estimations for selected papers.

Kinetic Models	Count	Parameter Estimation Method	Count
First-order kinetics	40	LOS plot	26
Michaelis-Menten kinetics	4	Lineweaver-Burk plot	3
Hyperbolic function	4	N/A	N/A
Parallel kinetics model	2	N/A	N/A
Sequential model	1	N/A	N/A
Peleg model	1	N/A	N/A
Weibull model	1	N/A	N/A
Quantification of starch fractions (RDS, SDS, RS)	16	N/A	N/A

**Table 6:** Statistical analysis for selected papers.

Statistical Analysis	Count
ANOVA (with follow-up tests)	41 (26)
correlation analysis	11
multivariate analysis	5
*t*-test	3
two-tailed test	2
statistical power analysis	1

## Data Availability

The data presented in this study are available in Supplementary Materials in the file Supplementary_data.xlsx.

## Supplementary Materials

The following are available online at https://www.mdpi.com/article/10.3390/foods11091223/s1.
